# Free Language Selection in the Bilingual Brain: An Event-Related fMRI Study

**DOI:** 10.1038/srep11704

**Published:** 2015-07-16

**Authors:** Yong Zhang, Tao Wang, Peiyu Huang, Dan Li, Jiang Qiu, Tong Shen, Peng Xie

**Affiliations:** 1 Institute of Neuroscience, Chongqing Medical University, Chongqing 400016, China; 2 Chongqing Key Laboratory of Neurobiology, Chongqing 400016, China; 3 Department of Neurology, the First Affiliated Hospital, Chongqing Medical University, Chongqing 400016, China; 4 School of Foreign Languages, Southwest University of Political Science and Law, Chongqing 401120, China; 5 School of Educational Science, Chongqing Normal University, Chongqing 400047, China; 6 School of Psychology, Southwest University, Chongqing 400715, China; 7 College of English Language and Literature, Sichuan International Studies University, Chongqing 400031, China

## Abstract

Bilingual speakers may select between two languages either on demand (forced language selection) or on their own volition (free language selection). However, the neural substrates underlying free and forced language selection may differ. While the neural substrates underlying forced language selection have been well-explored with language switching paradigms, those underlying free language selection have remained unclear. Using a modified digit-naming switching paradigm, we addressed the neural substrates underlying free language selection by contrasting free language switching with forced language switching. For a digit-pair trial, Chinese-English bilinguals named each digit in Chinese or English either on demand under forced language selection condition or on their own volition under free language selection condition. The results revealed activation in the frontoparietal regions that mediate volition of language selection. Furthermore, a comparison of free and forced language switching demonstrated differences in the patterns of brain activation. Additionally, free language switching showed reduced switching costs as compared to forced language switching. These findings suggest differences between the mechanism(s) underlying free and forced language switching. As such, the current study suggests interactivity between control of volition and control of language switching in free language selection, providing insights into a model of bilingual language control.

The ability to flexibly adjust one’s behavior to different tasks in everyday life involves both endogenous cognitive control on one’s own volition and exogenous cognitive control in response to an external stimulus^1^,^2^. A special case to exemplify this ability is language selection in the bilingual brain. In a bilingual context, the speaker exhibits an adept feat to select between two languages either on his/her own volition or on demand. Thus, studies of bilingual language selection may provide insights into theories of language control in the bilingual brain, but also theories of cognitive control in general.

Language selection (or control) refers to the cognitive mechanism that controls which language to select^3^. Though it remains a matter of controversy how bilinguals control their two languages for selection, the neural substrates underlying bilingual language control have been well addressed with language production tasks^3^,^4^,^5^,^6^,^7^,^8^,^9^ and comprehension tasks^10^,^11^,^12^. A qualitative review by Abutalebi and Green^13^ has proposed that bilinguals engage cognitive control networks for achieving language control. The neural evidence points to multiple neural regions of control, including the left prefrontal cortex, anterior cingulate cortex, caudate nucleus and bilateral supramarginal gyrus. In a quantitative meta-analysis of functional neuroimaging studies on bilingual cognitive control, Luk *et al.*^14^ have identified eight reliable activated regions, among which only the caudate and left prefrontal cortex overlap with those specified in the bilingual control model proposed by Abutalebi and Green^13^. Thus, both the qualitative and quantitative models pinpoint bilingual language control to the fronto-subcortical circuit.

Language selection may fall into two categories: free language selection and forced language selection. The former refers to selecting a language on one’s own volition, while the latter refers to selecting a language on an external demand. However, prior studies on bilingual language selection are characterized by “forced selection” in the sense that bilingual subjects in the experiment are required to select a language or switch into a language as directed by experimentally-provided cues^15^. Therefore, it is of interest to investigate how bilinguals control their two languages when they have freedom to select the language they intend to use.

Notably, research into free language selection has remained relatively scarce. Previous behavioral research has revealed that the option to use either language improves bilingual fluency^16^,^17^ and even brings performance benefits^18^. A recent behavioral study by Gollan and Ferreira has suggested differences between the mechanisms underlying free language switching and forced language switching^15^. Although several fMRI studies have examined the neural substrates underlying free word production in monolingual speakers^19^,^20^, reporting the engagement of the supplementary motor area (SMA), premotor area and prefrontal cortex, no brain imaging studies have addressed the neural substrates underlying free language selection in bilingual speakers. Traditionally, digit-naming^21^,^22^,^23^,^24^ and picture-naming^3^,^4^,^5^,^6^,^7^,^8^ paradigms have been adopted to investigate language switching. Moreover, the common approach to studying volitional control is to contrast it with stimulus-driven control^25^.

In the current study, we used a modified digit-naming switching paradigm to examine language selection in bilinguals, by contrasting free language switching with forced language switching. Chinese-English bilingual subjects underwent event-related functional magnetic resonance imaging (ER-fMRI) while performing digit-naming tasks either on demand under forced language selection condition or on their own volition under free language selection condition. For free selection tasks, we followed the volitional task switching paradigm introduced by Arrington and Logan^26^. According to this paradigm, the subjects were given the freedom to perform tasks with the instruction “to select the tasks equally often and in a random order”.

The first aim was to elucidate the neural substrates underlying free language selection. Work on volitional control suggested that the middle frontal cortex (MFC) was more active on free task choice trials than on forced trials^27^,^28^,^29^. Therefore, we predicted that the MFC would be engaged in free language selection. The second aim was to address the neural substrates that underlay language switching when bilinguals selected between two languages on their own volition as compared to when they did so on demand. Based on previous behavioral findings^15^, we predicted that the neural mechanisms underlying free and forced language switching might differ.

## Results

### Behavioral Results

The image and behavioral data from 16 subjects were analyzed. Wrong language use, wrong digit naming, naming emendation, ahead-of-time response, and extreme slow response (three SDs above the mean RT for each subject) were counted as response errors. The behavioral results showed a small bias towards language switching under free selection condition. Besides, there were lower error rates under free selection condition than under forced selection condition (Table S1).

**Figure 1:**
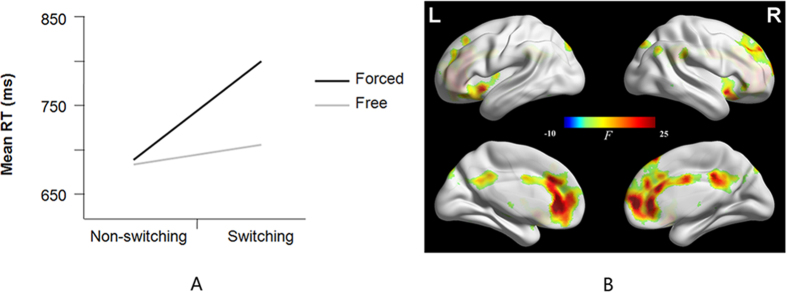
Two-Way ANOVA with Volition by Task Alternation. **A**. Interaction effect of response volition (free vs. forced) × task alternation (switching vs. non-switching) repeated-measures ANOVA. **B**. Volition (free vs. forced) × task alternation (switching vs. non-switching) interaction from the two-way ANOVA.

A response volition (free vs. forced selection) × task alternation (switching vs. non-switching) repeated-measures ANOVA revealed significant main effects of volition (F(1,15) = 96.45, *p* = 0.000) and task alternation (F(1,15) = 41.16, *p* = 0.000). There was also significant interaction (F(2,14) = 53.36, *p* = 0.000). Bonferroni’s test was used to correct the *post-hoc* tests and revealed a significant difference in naming latencies between free language switching and forced language switching (F(1,15) = 96.61, *p* = 0.000) but no significant difference in naming latencies between free language non-switching and forced language non-switching (F(1,15) = 0.38, *p* > 0.05). Free language selection (695 ms) was performed faster than forced language selection (741 ms). Free language switching (703 ms) took less time than forced language switching (798 ms), but free language non-switching (683 ms) took as much time as forced language non-switching (688 ms) (Fig. 1A).

For the free selection trials, data on naming latencies underwent a repeated-measures ANOVA with the factors of task alternation (switching, non-switching) and response language (L1 or Chinese, L2 or English). The results revealed significant main effects of task alternation (F(1,15) = 4.30, *p* < 0.05) and response language (F(1,15) = 18.04, *p* < 0.001). It took more time for language switching (703 ms) than for non-switching (683 ms) as well as for L2 naming (715 ms) than for L1 naming (671 ms). No interaction was found (F(2,14) = 0.01, *p* > 0.05). For the forced selection trials, the same measure was adopted. The results revealed significant main effects of task alternation (F(1,15) = 158.09, *p* < 0.001) and response language (F(1,15) = 7.61, *p* < 0.05). It took more time for language switching (798 ms) than for non-switching (688 ms) as well as for L2 naming (753 ms) than for L1 naming (727 ms). No interaction was found (F(2,14) = 1.64, *p* > 0.05). Apparently, free language switching (20 ms) displayed reduced switching costs as compared to forced language switching (110 ms).

For the direction of language switching, naming latencies were estimated using a two-sample *t*-test. There was no significant difference (t(1,15) = 1.63, *p* > 0.05) between freely switching into L2 (free forward switching) (726 ms) and L2 non-switching (704 ms) nor between freely switching into L1 (free backward switching) (681 ms) and L1 non-switching (661 ms) (t(1,15) = 1.31, *p* > 0.05). However, there were significant differences (t(1,15) = 7.34, *p* < 0.001) between forcedly switching into L2 (forced forward switching) (803 ms) and L2 non-switching (702 ms) as well as between forcedly switching into L1 (forced backward switching) (793 ms) and L1 non-switching (660 ms) (t(1,15) = 10.58, *p* < 0.001). The magnitude of switching costs was larger for forcedly switching into L1 (backward switching or L2-to-L1: 133 ms) than for forcedly switching into L2 (forward switching or L1-to-L2: 101 ms). However, there was almost no difference between freely switching into L1 (backward switching or L2-to-L1: 20 ms) and freely switching into L2 (forward switching or L1-to-L2: 22 ms).

The verbal response time for the first digit and the second digit was respectively defined as RT1 and RT2. The difference in RT1 and RT2 was treated as an indicator of facilitation effects (reduced naming latencies in RT2 relative to RT1). Significant facilitation effects for switching (t(1,15) = 2.73, *p* < 0.05, mean RT1 = 811 ± 122 ms and RT2 = 704 ± 118 ms) and for non-switching (t(1,15) = 2.58, *p* < 0.05, mean RT1 = 799 ± 123 ms and RT2 = 683 ± 107 ms) were found under free selection condition. There were no significant facilitation effects under forced switching condition (t(1,15) = 0.49, *p* > 0.05, mean RT1 = 771 ± 117 ms and RT2 = 798 ± 129 ms) nor under forced non-switching condition (t(1,15) = 1.98, *p* > 0.05, mean RT1 = 740 ± 127 ms and RT2 = 688 ± 90 ms).

### fMRI Results

#### Free Language Selection (Free Language Switching and Non-switching)

**Figure 2:**
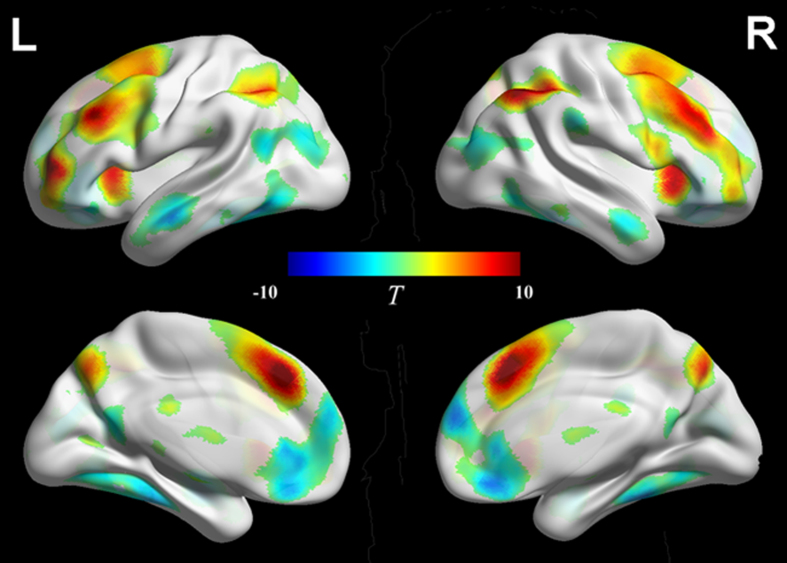
Comparison of Free Language Selection and Forced Language Selection. Activations depicted in “hot” colors represent regions that were significantly more active under free language selection condition compared to forced language selection condition. Activations depicted in “cold” colors represent regions that were significantly more active under forced language selection condition compared to free language selection condition. *p* < 0.001, k > 50 voxels, FDR-corrected.

A two-way ANOVA with volition (free, forced) by task alternation (switching, non-switching) as the stimulus conditions revealed a main effect of volition, a main effect of task alternation, and an interaction between volition and task alternation. The interaction engaged the bilateral anterior cingulate cortex (BA 32) and superior medial frontal gyrus (BA 10) (Table 1 and Fig. 1B). Volition engaged the frontoparietal regions, including the bilateral supplementary motor area (BA 6), superior/middle frontal gyrus (BA 8/9/46), inferior frontal gyrus and operculum/ triangle (BA 10/11/44/45/47), anterior cingulate cortex (BA 32), superior parietal lobule (BA 7), supramarginal gyrus (BA 40), caudate, insula, thalamus, putamen and cerebellum crus1 (Table 1 and Fig. 2). Task alternation engaged the bilateral pre-central and post-central gyrus (BA 2/3/4), middle frontal gyrus (BA 6), thalamus and left superior parietal lobe (BA 7) (Table 1).

Furthermore, we examined the neural substrates underlying free language switching and non-switching. Free language switching relative to forced language switching showed activations in the bilateral supplementary motor area (BA 6), superior/middle frontal gyrus (BA 8/9/46), inferior frontal gyrus (BA 10/13/47), middle cingulate cortex (BA 32), superior parietal lobule (BA 7), supramarginal gyrus (BA 40) and insula. However, no significant activation was found for forced language switching relative to free language switching (Table S2 and Fig. S1A). Free language non-switching relative to forced language non-switching showed activations in the bilateral supplementary motor area (BA 6), superior/middle frontal gyrus (BA 8/9/46), inferior frontal gyrus (BA 10/13/47), cingulate cortex (BA 32), superior parietal lobule (BA 7), supramarginal gyrus (BA 40) and insula. Forced language non-switching relative to free language non-switching showed activations in the bilateral superior/medial frontal gyrus (BA 9/10), anterior cingulate cortex (BA 32), fusiform gyrus (BA 36/37), middle temporal gyrus (BA 39), middle occipital gyrus (BA 19) and right supramarginal gyrus (BA 40) (Table S2 and Fig. S1B).

#### Language Switching

**Figure 3:**
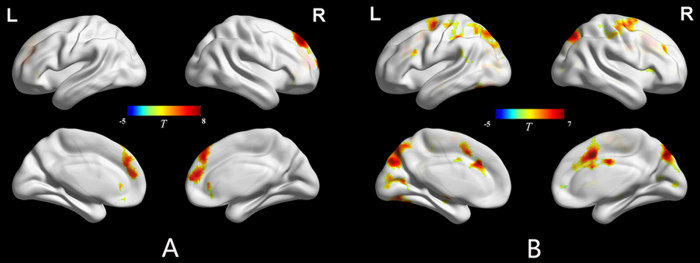
Comparisons of Language Switching and Non-Switching. **A**. Free language switching vs. free language non-switching. **B**. Forced language switching vs. forced language non-switching. Activations depicted in “hot” colors represent regions that were significantly more active during language switching compared to non-switching. Activations depicted in “cold” colors represent regions that were significantly more active during language non-switching compared to language switching. *p* < 0.001, k > 50 voxels, FDR-corrected.

To examine the neural substrates underlying language switching, we contrasted language switching with non-switching. Free language switching relative to free non-switching showed activations in the bilateral middle/medial frontal gyrus (BA 8/9), anterior cingulate cortex (BA 32), inferior frontal gyrus (BA 10/11), left supplementary motor area (BA 6) and right inferior parietal lobule (BA 39/40) (Table 2 and Fig. 3A). Forced language switching relative to forced non-switching showed activations in the bilateral supplementary motor area (BA 6), cingulate cortex (BA 32), middle/inferior frontal gyrus (BA 45/46), thalamus, putamen, left fusiform gyrus and pre-cuneus/cuneus (BA 18/19), left superior parietal lobule (BA 7), left supramarginal gyrus (BA 40), left hippocampus, right insula and caudate (Table 2 and Fig. 3B). However, both free non-switching relative to free language switching and forced non-switching relative to forced language switching showed no significant activation. In addition, the conjunction analysis of free language switching relative to non-switching and forced language switching relative to non-switching revealed no significant activation.

#### Direction of Language Switching

To examine whether the activation patterns were different regarding the direction of language switching, we compared switching into L2 (forward switching) with L2 non-switching and switching into L1 (backward switching) with L1 non-switching. Free backward switching relative to free L1 non-switching showed activations in the bilateral superior frontal gyrus (BA 6), medial frontal gyrus (BA 9/10) and anterior cingulate cortex (BA 32). Free L1 non-switching relative to free backward switching showed activations in the left pre-central/post-central gyrus (BA 3/4) and left inferior parietal lobule (BA 40) (Table S3 and Fig. S2A). No significant activation was found for free forward switching relative to free L2 non-switching and vice-versa. Forced backward switching relative to forced L1 non-switching showed activations in the bilateral supplementary motor area (BA 6), middle cingulate cortex (BA 32), inferior parietal lobule (BA 40), left fusiform gyrus and pre-cuneus (BA 18/19), and left pre-central/post-central frontal gyrus (BA 3/4). Forced L1 non-switching relative to forced backward switching showed activations in the right pre-central/post-central gyrus (BA 3/4) (Table S3 and Fig. S2B). No significant activation was found for forced forward switching relative to forced L2 non-switching and vice-versa. In addition, we found no significant activations for free forward switching versus free backward switching and vice-versa nor for forced forward switching versus forced backward switching and vice-versa.

## Discussion

In the current study, we first addressed the neural substrates underlying free language selection (both free language switching and non-switching) and found the active engagement of the frontoparietal regions under free language selection condition. Furthermore, we examined the differences between the mechanism(s) underlying free and forced language switching. The results demonstrated differences not only in the pattern of brain activation but also in language switching costs. Additionally, the direction of language switching was considered. Below we will discuss how our findings fit with the extant literature on volitional control and language switching and what discrepancies there are between the findings in the current study and those in prior studies.

The main effect of volition in the current study was found to engage the frontoparietal regions, i.e., the supplementary motor area (SMA), anterior cingulate cortex (ACC), premotor cortex (PMC), dorsolateral prefrontal cortex (DLPFC), ventrolateral prefrontal cortex (VLPFC) and posterior parietal cortex (PPC). The activation in those regions may reflect the engagement of cognitive control functions under free language selection condition. Arrington and Logan^26^ have introduced the voluntary task switching (VTS) paradigm and assumed that the requirement to select a task in the absence of an external cue would involve cognitive control. In a study on voluntary language switching, Gollan and Ferreira^15^ have suggested that voluntary language switching engages more efficient cognitive control than involuntary language switching. These suggestions fit well with the frontoparietal function which involves general cognitive control. Neuroimaging studies have offered further supportive evidence. Forstmann *et al.*^29^ compared voluntary task selection to forced task selection and found that the middle frontal cortex (MFC) were active on both voluntary and forced trials as well, but to a greater extent on voluntary task selection trials. They suggested that the MFC was involved in resolving uncertainty between the different task alternatives. In a recent study on voluntary task selection in the VTS paradigm, Orr and Banich^30^ also found more activation in the MFC on voluntary selection trials than on forced selection trials. They suggested that cognitive control mechanisms were engaged in selecting the appropriate task under the voluntary condition and concluded that the MFC was critical in voluntarily choosing between different response alternatives.

In the “what, when, whether” model^27^ of volitional behavior, Brass and Haggard have proposed that within the MFC, the ACC mediates the “what component” (i.e., the decision about which action to execute), while the pre-SMA/SMA mediates the “when component” (i.e., the decision about when to execute an action), and the dorsal fronto-median cortex is involved in the “whether component” (i.e., the decision about whether to execute an action or not). Neuroimaging^31^ and single neuron recording^32^ studies on volitional act have pinpointed the role of the SMA in the planning of the self-generated behavior. Neuroimaging studies^2^,^29^,^30^ on free response selection have showed elevated activation in the ACC under conditions where subjects could freely select a movement or response set. It has been reported that free language production in monolinguals engages the MFC as well. The comparison of internally and externally specified selection of verbal response in monolinguals has revealed strong activity in the left DLPFC and VLPFC, as well as the medial frontal cortex^19^,^20^. The VLPFC has also been found to be activated in such non-linguistic cognitive tasks as volitional actions in patients^33^ and endogenous preparation without an external stimulus^1^.

Apart from the MFC, the parietal cortex was active on free selection trials in the current study. The parietal cortex, historically assumed to be a sensory structure, is now viewed as an area important for the formation of intentions^31^,^34^,^35^. Studies from lesions in patients^36^,^37^ have also provided supportive evidence for the role of the posterior parietal cortex (PPC) in volitional control of action. An alternative interpretation is that the PPC may be associated with volitional orienting of attention^38^,^39^.

Nevertheless, there appears a discrepancy between the findings in the current study and those in the study by Orr and Banich^30^. Orr and Banich have suggested a dissociation of the mechanisms underlying voluntary task selection, with the frontal pole underlying task selection guided by internal goals, and the MFC underlying task selection guided by the weighing of different response alternatives. But no activation in the frontal pole was found under free language selection condition in our study. One possible account for the discrepancy is that different brain regions are involved in the actual execution of the specific voluntary action^35^. A case in point is that volitional motor action engages the motor system, including the SMA and bilateral primary motor cortices^32^,^34^; In the case of volitional abstract mental task, the angular gyrus is engaged in the retrieval of abstract facts from memory^35^,^40^. However, it remains unclear what exact portion of the MFC is engaged in free language selection tasks.

Our results showed that both free language switching and forced language switching engaged prefrontal-parietal-subcortical regions though not overlapping regions. These finding are compatible with Abutalebi and Green’s cognitive model of bilingual language control^13^. According to this model, the prefrontal cortex, anterior cingulate cortex, caudate and supramarginal gryus are key regions which mediate bilingual language control. Using a meta-analysis, Luk *et al.*^14^ reported that the caudate and left prefrontal cortex overlapped with those specified in Abutalebi and Green’s bilingual control model.

The extant literature of language switching has shown that the frontoparietal network serves the language control function in bilingual speakers. The middle frontal cortex (MFC) and inferior frontal cortex (IFC) have been found to be engaged in bilingual language switching and selection^3^,^4^,^5^,^6^,^8^. The SMA shows evidence of playing a role in inhibiting non-target language during language switching^6^,^41^ or selecting words between languages^42^,^43^. The ACC has been found to be involved in selecting the target language, monitoring errors, and detecting conflicts in language switching^3^,^4^,^5^,^8^. The engagement of the supramarginal gyrus in language switching may reflect the increased demands for phonological recording^7^,^8^,^12^,^44^,^45^, while the superior parietal lobule may play a role in reconfiguring stimulus-response mappings^46^,^47^.

However, the conjunction analysis of free language switching and forced language switching revealed no significant activation of overlapping regions, suggesting differences between the neural mechanisms underlying them. Free language switching in our study revealed a similar pattern of activation to that of free task selection in Orr and Banich’s study^30^. Meanwhile, forced language switching in our study revealed bilateral activation in the frontoparietal regions, consistent with prior studies on forced language switching^3^,^4^,^5^,^6^,^7^,^8^. Arrington and Logan assumed that volitional task switching engaged more efficient cognitive control than explicit task switching^26^.

Both free language switching (relative to free non-switching) and forced language switching (relative to forced non-switching) showed switching costs, consistent with Green’s inhibitory control model^48^. According to this model, language switching involves a language control mechanism which allows bilinguals to select the target language while inhibiting the interferences from the non-target language. Inhibition takes time and yields a switching cost. Of note, free language switching showed reduced switching costs as compared to forced language switching in the current study. The behavioral study on volitional language switching by Gollan and Ferreira^15^ also reported that volitional language switching was significantly slower than volitional language non-switching. Nonlinguistic switching costs have been reported to be reduced but not eliminated when switches are predictable^49^ or volitional^26^,^50^. It is suggested that bilinguals may apply as little as is needed when they do apply inhibition^15^,^51^ during free language switching.

Concerning the direction of language switching, it is well established that unbalanced bilinguals exhibit asymmetric switching costs (i.e., greater costs during backward switching than during forward switching)^23^. However, unbalanced bilinguals in the current study showed symmetric switching costs for free language switching, consistent with the finding in the study by Gollan and Ferreira (Experiment 1)^15^. Meanwhile, they showed asymmetric switching costs for forced language switching, in line with the findings of prior studies which investigated (forced) language switching with digit-naming paradigm^23^,^24^ and picture-naming paradigm^8^.

The switch-cost asymmetry has been interpreted as the signature of inhibitory control of the non-target language^52^. Symmetric switching costs have been reported for highly proficient bilinguals^53^. Does the absence of switch-cost asymmetry suggest no inhibitory control for free language switching? By Gollan and Ferreira’s account^15^, free language switching might allow unbalanced bilinguals to function more like highly proficient bilinguals such that unbalanced bilinguals might apply inhibition both on switching and on non-switching trials under volitional instruction, yielding symmetric switching costs.

Using a picture-naming paradigm, Wang *et al.* investigated the neural bases of asymmetric language switching and reported more activation of brain regions for forward switching than for backward switching^8^. However, the current study found no significant activation of brain regions when forward switching was compared with backward switching and vice versa under free language selection condition. The same held true for forced language selection condition. We propose that the differences between the current results and those of Wang and colleagues derive from differences in the experimental designs. The trial in our study involved a digit pair such that it reflected the effect of within-trial switching, whereas the trial in their study was a single picture such that it reflected the effect of between-trial switching.

We failed to replicate the “unusual findings” which were reported in Gollan and Ferreira’s study^15^. Within the fifty-percent condition under which bilingual subjects were required to use either language equally often, Gollan and Ferreira reported staying (non-switching) costs rather than switching costs — “highly unusual results”. They explained the “unusual findings” as the results of different levels of task difficulty. Specifically, the subjects in other prior studies switched between two tasks of approximately equal difficulty^26^,^50^, whereas L1 was apparently dominant over L2 in their study such that bilinguals would choose to stay in L1 when it was difficult for them to switch into L2. Thus, the staying (non-switching) trials would involve highly costly controlled processes. By comparison, digit naming is a more automatic process and may not require much involvement of cognitive control^24^. In our study, digit naming in either language was a task with relatively comparable difficulty such that the subjects did not have to avoid difficult trials. Further studies are needed to address whether free language switching would exhibit “unusual staying costs” in unbalanced bilinguals with more controlled levels of task difficulty.

Here, Chinese-English bilinguals under free language selection condition switched between languages a little more than they stayed in both languages. Gollan and Ferreira^15^ also reported that switching rates were lower than non-switching rates in Experiment 1 (voluntary or either-language condition), whereas switching rates increased to 52% in Experiment 2 (quasi-voluntary or fifty-percent condition). Thus, Gollan and Ferreira assumed that bilinguals used qualitatively different strategies under either-language condition and fifty-percent condition. To meet the requirements of the fifty-percent instruction, bilinguals switched languages more often such that switching trials outnumbered staying trials in both languages. Besides, performance benefits that bilingual subjects gained from the option to freely select tasks^15^,^16^,^54^ may be another possible account for higher switching rates. On the other hand, our behavioral results showed longer reaction times and higher error rates under forced language selection condition than under free language selection condition. Before the presentation of the second cue, the response under free language selection condition was goal-directed and thus certain while the response under forced language selection condition was stimulus-driven and thus uncertain. As a result, this uncertainty might introduce conflict^55^, leading to longer reaction times and higher error rates when language selection was not volitional.

Here it is necessary to note the seemingly contradictory results between the behavioral data and fMRI data, i.e., free language switching as compared to forced language switching showed reduced switching costs but engaged more cognitive control. The discrepancy might be owed to the nature of being volitional. In the studies on volitional actions, Soon and colleagues^34^,^35^ have found that the human brain may start preparing spontaneous movements several seconds before the participant reports they are consciously making a decision to move. In the case of free language switching, it is possible that the response time reflects the result of having switched whereas the fMRI data reflect the processing of volitional decision and language switching. As a consequence, free language switching showed less response time but more cognitive control than forced language switching. Orr and Banich^30^ also reported that both voluntary and explicit trials recruited cognitive control regions, but to a greater degree during voluntary task choices than during explicit tasks, while voluntary trial took less time to respond than explicit trials. However, as volition involves a set of decision processes in the specific brain circuits^25^, it is hard to offer a convincing account here.

In conclusion, volitional control is one of the key components when we address the neural substrates of bilingual language control which occurs in a natural context. As such, we suggest that free language selection engages interactivity between control of volition and control of language switching. Under natural conditions, language switching in bilingual speakers is rather individualized and context-dependent. Although Abutalebi and Green’s bilingual language control model^13^ and Green’s inhibitory control model^48^ have attempted to account for the mechanisms underlying free language control in day-to-day life, it remains difficult to experimentally capture the conditions of natural human volition by instructing subjects to be volitional – an obviously paradoxical situation^25^. Although the experimental conditions of the current study are not quite satisfactory, the internally-initiated versus externally-exerted comparison employed here provides a model to investigate free language selection (or control) in the human brain.

## Methods

### Subjects

A total of 22 right-handed Chinese-English bilingual subjects were initially recruited. We obtained written informed consent from each subject in accordance with the guidelines approved by the Research Ethical Committee of Southwest University (Chongqing, China). All subjects had normal or corrected-to-normal vision. No subject had a history of major medical, neurological, or psychiatric disorder. The data of six subjects were excluded from the analysis due to task-related issues (malfunctioning buttons and/or inability to follow task instructions to perform the tasks equally often) during the fMRI session: two subjects were excluded due to high error rates (>5%) in response under forced language selection condition, and four more subjects were excluded due to low response rates (<60%) under free language selection condition (i.e., the number of response rates under any one of the four free selection conditions was less than 60% of the average). Therefore, the final sample consisted of 16 subjects (7 males, 9 females, mean age = 21.3 ± 2.38).

### Language Proficiency Assessment

All subjects were Chinese-English bilinguals who majored in English language and literature from at the College of International Studies, Southwest University (Chongqing, China). All subjects were educated in China and started learning English as their second language in junior high school at the age of 11.9 ± 1.2 (age of acquisition, AoA). As was conducted by Krizman and colleagues^56^, we assessed the subjects’ language proficiency in English and Chinese through the Language Experience and Proficiency Questionnaire (LEPQ)^57^ with a slightly-adapted Chinese version. Their English proficiency ratings were 8.4 ± 0.5 in speaking English, 8.6 ± 0.6 in comprehending spoken English, and 8.9 ± 0.5 in reading English, while their Chinese proficiency ratings were 9.2 ± 0.4 in speaking Chinese, 9.3 ± 0.4 in comprehending spoken Chinese, and 9.5 ± 0.3 in reading Chinese. Furthermore, we assessed the subjects’ English proficiency on the basis of the Test for English Majors Band 4 (TEM4). All subjects passed the TEM4 (mean percentage = 75% ± 5.6%) with a writing score of 71% ± 5.2%, a reading score of 79% ± 6.2%, and a listening score of 77% ± 5.7%. Table S4 listed the scores of the 16 subjects’ self-ratings and proficiency tests.

### Stimuli

**Figure 4:**
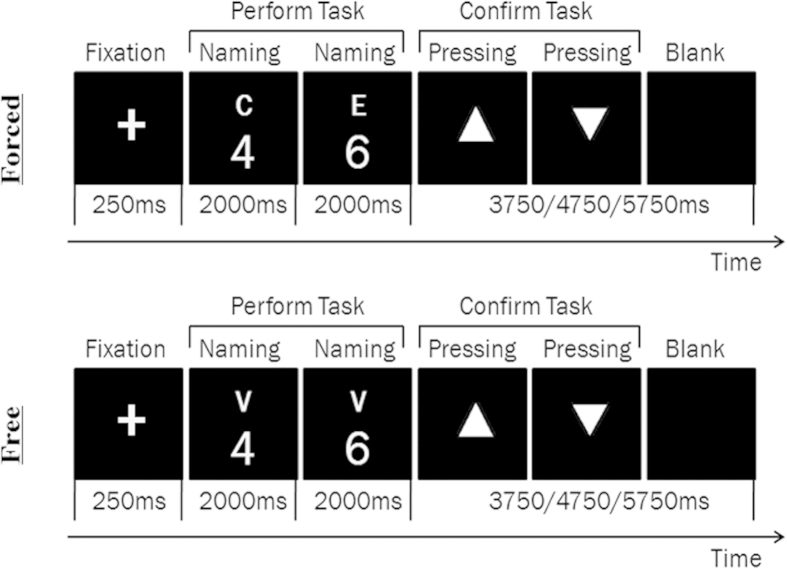
Examples of a Forced Language Selection Trial (Top Panel) and a Free Language Selection Trial (Bottom Panel). Each trial was a digit pair involving a task performance (i.e., digit-naming) followed by a task confirmation (i.e., button-pressing). Each trial began with a 250-ms fixation cross. Then a digit (placed at the bottom in a larger font) with a cue (placed at the top in a smaller font) was visually presented for 2000 ms followed by the second digit with a cue presented for 2000 ms. During task performance, subjects named each digit in Chinese or English according to the cue (“C” and “E” were the cues for forced selection trials, and “V” was the cue for free selection trials). For forced selection trials, subjects named each digit on demand (“C” for Chinese and “E” for English). For free selection trials, subjects named each digit on their own volition (“V” for Chinese or English) with the instruction to select the tasks (four free selection conditions: CC, CE, EE, and EC) equally often and in random order. Afterwards, two different triangles were successively presented and served as the cues for button-pressing confirmation. During task confirmation, subjects pressed buttons successively with their left or right thumb to confirm their responses by self-monitoring (a left thumb pressing for English or a right thumb pressing for Chinese). The triangle presentation terminated upon button-pressing, followed by a blank screen. The total time for task confirmation and the blank screen was either 3750, 4750, or 5750 ms.

An ER-fMRI design was used. There were two types of language selection: forced and free language selection. Each trial was composed of two successively-presented digits (digit-pair) (Fig. 4). For a digit-pair trial, the subjects were asked to name each digit (1–9) in Chinese or English either (i) on demand under forced selection condition or (ii) on their own volition under free selection condition, depending on the cue type. Three cues were used with “E” (English) and “C” (Chinese) for forced language selection and “V” (volitional) for free language selection. Thus, the digit pairs fell into four conditions for either free language selection (CC, CE, EE and EC) or four conditions for forced language selection (CC, CE, EE and EC). The CE and EC conditions were defined as “language switching”, while the CC and EE conditions as “non-switching”. The nine digits made up 72 unique digit-pairs. There were four different runs with 72 trials in each run. Free and forced selection trials were presented in a random order throughout the experiment. The trial sequences in each run were jittered and optimized using the GA algorithm^58^. Instructions for free language selection were modeled on the task instructions published by Arrington and Logan^26^, which required the subjects to select the tasks equally often and in a random order.

Each trial began with a 250-ms fixation cross. Then, subjects were required to perform tasks of digit-naming. The first digit (placed at the bottom in a larger font) and cue (placed at the top in a smaller font) was visually presented for 2000 ms, followed by the second digit and cue presented for 2000 ms. Subjects were instructed to name each digit as accurately and quickly as possible in a soft voice according to the cue without moving their heads^59^. To qualitatively control the subject’s verbal responses, they were informed that their responses were continuously recorded using an interphone device^3^. After performing two digit-naming tasks, subjects were instructed to confirm the tasks by button-pressing. Two different triangles were successively presented and served as cues for button-pressing. Half of the subjects self-monitored their language responses with a left thumb pressing for English and a right thumb pressing for Chinese (e.g., left-left for EE and left-right for EC). The other half of the subjects self-monitored their language responses with a left thumb pressing for Chinese and a right thumb pressing for English. The triangle presentation terminated upon button-pressing, followed by a blank screen. The total time for the task-confirming and blank screen was either 3750, or 4750, or 5750 ms.

As the task confirmation procedure may have contaminated the effects of between-trial language switching, but not the effects of within-trial language switching, we took a digit-pair trial as a mini-block and only considered within-trial language switching in our analysis.

### Procedure

The experiment consisted of three sessions: a practice session, an fMRI session and a behavioral session. A demo program with a run of 72 digit-pair trials was conducted as practice. First, the subjects practiced digit-naming according to the cue (task-performing) and button-pressing according to self-monitoring responses (task-confirming). Second, following the task instruction by Arrington and Logan^26^, the subjects practiced freely performing language tasks (four free selection conditions: CC, CE, EE and EC) with the instruction “to select the tasks equally often and in a random order”. If the proportion of any one of the four free selection conditions was less than 60% of the average, or the accuracy fell below 90%, the practice was repeated as recommended by Orr and Banich^30^. After the practice session, the subjects underwent four runs in the scanner during fMRI session. A short break was provided between runs. Afterwards, the subjects underwent four identical runs outside the scanner during the behavioral session as performed by prior studies on language switching with production tasks^3^,^4^,^6^,^8^,^24^.

### fMRI Data Acquisition

Functional MRI scans were acquired with a Siemens 3.0 T Trio scanner at the MR Research Center of Southwest University. Stimuli were programmed with E-prime software 2.0 (Psychology Software Tools, Inc., Pittsburgh, PA) and projected onto a translucent screen via a projector. The subjects viewed the stimuli through a mirror attached to the head coil. Head motion was minimized by placing pillows and cushions around the head. Ear plugs were used to reduce noise in the scanner. Functional imaging consisted of an echo planner imaging with gradient echo (EPI GRE) sequence (TR = 2000 ms, TE = 30 ms, flip angle = 90°, FOV = 64 × 64 mm^2^, 32 interleaved descending slices, voxel size = 3.44 × 3.44 × 3.00 mm^2^ with 1-mm intra-slice gap). The five initial scans of each session were dummy scans that were used to equilibrate the state of magnetization and were excluded from the analysis. Anatomical reference images, acquired after the functional imaging, consisted of a 3-D GRE T1-weighted sequence (TR = 1900 ms, TE = 2.52 ms, flip angle = 9°, FOV = 256 × 256 mm^2^, voxel size = 1 × 1 × 1 mm^3^). Head motion was evaluated on the MRI workstation as soon as the scans ended.

### Image Processing and Statistical Analysis

Data processing and statistical analyses were analyzed using spm8 (Wellcome Trust Centre for Neuroimaging, London, UK, http://www.fil.ion.ucl.ac.uk/spm/) and implemented on MATLAB version 2010a. All functional volumes were first corrected for slice timing with the middle slice as reference, and then realigned to remove head motion. Functional images were brought into the MNI space by applying parameters of normalizing co-registered anatomical images. Resampled 3-mm isometric images were smoothed using an 8-mm full width at half maximum Gaussian kernel. Time-series from each voxel were high-pass filtered (1/128 Hz cutoff) to remove low-frequency noise and signal drift. At the first level, the preprocessed functional volumes were submitted to fixed-effects analyses for each subject, with the general linear model applied at each voxel across the whole brain. Within-subject hemodynamic responses for each condition were assessed to generate statistical parametric maps for each specified condition, including the four free selection conditions (CC, CE, EE and EC), the four forced selection conditions (CC, CE, EE and EC) and the four conditions with volition (free vs. forced) by task alternation (switching vs. non-switching) as the stimulus conditions. Six parameters of head motion were regressed out as nuisance variables. Group level random-effects analyses were performed using one-sample *t*-tests for first-level contrasts of interest.

A two-way repeated-measures ANOVA was also conducted for the main effects of volition/ task alternation and their interaction. We further conducted *post hoc t*-test for free language switching versus forced language switching and free language non-switching versus forced language non-switching. To examine the differences between the mechanisms underlying free and forced language switching, we compared free language switching versus free language non-switching, and forced language switching versus forced language non-switching. To examine whether there was activation of overlapping regions between free and forced language switching, we conducted a conjunction analysis of free language switching versus free language non-switching and forced language switching versus forced language non-switching. To further consider the direction of language switching, we conducted the following comparisons: free switching into L1 versus free L1 non-switching; free switching into L2 versus free L2 non-switching; forced switching into L1 versus forced L1 non-switching; forced switching into L2 versus forced L2 non-switching. To assess whether there was asymmetric switching, we compared: free forward switching (switching from L1 to L2) versus free backward switching (switching from L2 to L1) and forced forward switching versus forced backward switching. All of the resulting statistical maps were set to a significant threshold of *p* < 0.001 (false discovery rate (FDR)-corrected)^60^. An extent threshold of 50 contiguous voxels was applied to all contrasts. Figures were visualized by Brain-Net Viewer^61^.

## Additional Information

**How to cite this article**: Zhang, Y. *et al.* Free Language Selection in the Bilingual Brain: An Event-related fMRI Study. *Sci. Rep.* **5**, 11704; doi: 10.1038/srep11704 (2015).

## Supplementary Material

Supplementary Information

## Figures and Tables

**Table 1:** Activated Clusters from 2 × 2 ANOVA with Volition by Task Alternation*.

**Region**	**Clustersize**	**BA areas**	**T/Fvalue**	**MNI coordinates**		
				x	y	z
*Volition x Task alternation*						
L/R anterior cingulate cortex	357	10 32	41.53	3	51	3
L/R superior medial frontal gyrus						
*Volition*						
L/R superior/middle frontal gyrus	4479	6 8 9 10 32 44 45 46 47	12.17	0	30	42
L/R supplementary motor area						
L/R inferior frontal gyrus						
L/R anterior cingulate cortex						
L/R caudate/putamen/insula						
R supramarginal parietal lobule	756	7 39 40	12.47	45	−45	48
R superior parietal lobule						
R angular						
R inferior frontal gyrus	639	44 45 47	9.64	33	21	−3
L superior /inferior parietal lobule	599	7 40	9.44	−42	−54	48
L angular						
R superior parietal lobule	398	7	7.98	9	−72	45
R cerebellum crus1	82	n/a	6.44	36	−63	−33
L cerebellum crus1	76	n/a	5.87	−33	−63	−33
*Task alternation*						
R pre-/post-central gyrus	909	3 4 6	8.28	33	−12	60
R middle frontal gyrus						
L pre-/post-central gyrus	602	3 4 6	7.60	−24	−3	21
L supplementary motor area						
L superior parietal lobe	189	7	6.46	−12	−66	51
L thalamus	136	n/a	5.99	−15	−21	0
R thalamus	127	n/a	5.49	15	−21	6

^*^p < 0.001, k > 50 voxels, FDR-corrected

**Table 2:** 

**Region**	**Cluster size**	**BA areas**	**T value**	**MNI coordinates**		
				**x**	**y**	**z**
*Free switching vs. free non-switching*						
L/R middle/inferior frontal gyrus	1827	6 8 9 10 11 32	10.84	15	42	42
L/R medial frontal gyrus						
L/R anterior cingulate cortex						
L supplementary motor area						
R inferior parietal lobule	79	39 40	6.04	48	−66	36
***Forced switching vs. forced non-switching***						
L superior parietal lobule	4026	6 7 1819 24 32	9.38	−39	−60	−15
L fusiform gyrus						
L precuneus/cuneus						
L/R superior/middle occipital gyrus						
L/R supplementary motor area						
L/R cingulate cortex						
L/R thalamus	939	13	7.57	−9	−12	−12
L/R putamen						
L hippocampus						
R insula						
R caudate						
R superior/middle frontal gyrus	624	6	10.57	33	−18	57
R supplementary motor area						
L middle/inferior frontal gyrus	105	45 46	5.81	−39	21	15
R medial/orbital frontal gyrus	70	10 32	5.01	15	54	0
R anterior cingulate cortex						
L supramarginal gyrus	51	40	4.83	−57	−42	27

^Table 2. Activated Brain Regions when Contrasting Language Switching with Language Non-Switching*.*^p < 0.001, k > 50 voxels, FDR-corrected
