# Supplementary material for: Free Language Selection in the Bilingual Brain: An Event-Related fMRI Study
Source: Sci Rep. 2015 Jul 16;5:11704. doi: 10.1038/srep11704 (PMC4503947; doi:10.1038/srep11704)
Supplement: Supplementary Information [file srep11704-s1.doc]

**Free Language Selection in the Bilingual Brain: an Event-Related fMRI Study**

**(Supplementary Materials)**

Yong Zhang^1,2,4*^, Tao Wang^1,2*^, Peiyu Huang^1,2^, Dan Li^1,2,5^, Jiang Qiu^1,2,6^, Tong Shen^7^, & Peng Xie^1,2,3↑^

^1^ Institute of Neuroscience, Chongqing Medical University, Chongqing 400016, China

^2^ Chongqing Key Laboratory of Neurobiology, Chongqing 400016, China

^3^ Department of Neurology, the First Affiliated Hospital, Chongqing Medical University, Chongqing 400016, China

^4^ School of Foreign Languages, Southwest University of Political Science and Law, Chongqing 401120, China

^5^ School of Educational Science, Chongqing Normal University, Chongqing 400047, China

^6^ School of Psychology, Southwest University, Chongqing 400715, China

^7^ College of English Language and Literature, Sichuan International Studies University, Chongqing 400031, China

*These authors contributed equally to this work.

^↑^***Corresponding author:***

Professor Peng Xie

Department of Neurology

The First Affiliated Hospital, Chongqing Medical University

1 Youyi Road, Yuzhong District, Chongqing 400016, China

Phone: +86-23-68485490

Fax: +86-23-68485111

Email: [xiepeng@cqmu.edu.cn](mailto:xiepeng@cqmu.edu.cn)

**Supplementary Materials**

**Tables (Supplementary, Total 4)**

**Table S1. Naming Frequency, Errors, mean RTs and corresponding SDs under Free and Forced Selection Conditions in fMRI and Behavioral Sessions**

|  | **fMRI Session** | | | |  | **Behavioral Session** | | | |
| --- | --- | --- | --- | --- | --- | --- | --- | --- | --- |
|  | **CC** | **CE** | **EE** | **EC** |  | **CC** | **CE** | **EE** | **EC** |
| ***Free selection*** |  |  |  |  |  |  |  |  |  |
| Naming frequency | 25.3%  (2.4%) | 25.8%  (2.6%) | 21.5%  (2.2%) | 27.3%  (2.8%) |  | 22.9%  (2.3%) | 26.1%  (2.6%) | 26.7%  (2.6%) | 24.3%  (2.5%) |
| Errors | 2.3% | 2.9% | 3.1% | 3.2% |  | 2.5% | 2.8% | 3.4% | 3.6% |
| Mean RTs (SDs) |  |  |  |  |  | 661 ms  (87 ms) | 726 ms  (109 ms) | 704 ms  (96 ms) | 681 ms  (91 ms) |
| ***Forced selection*** |  |  |  |  |  |  |  |  |  |
| Naming frequency | 25% | 25% | 25% | 25% |  | 25% | 25% | 25% | 25% |
| Errors | 3.4% | 3.8% | 4.0% | 4.1% |  | 3.6% | 4.1% | 4.1% | 4.3% |
| Mean RTs (SDs) |  |  |  |  |  | 660 ms  (83 ms) | 803 ms  (126 ms) | 702 ms  (101 ms) | 793 ms  (124 ms) |

**Table S2. Activated Brain Regions during Free Language Switching Versus Forced Language Switching and Free Language Non-Switching Versus Forced Language Non-Switching***

| **Region** | **Cluster size** | **BA**  **areas** | **T**  **value** | **MNI coordinates** | | |
| --- | --- | --- | --- | --- | --- | --- |
|  |  |  |  | **x** | **y** | **z** |
| ***Free switching vs. forced switching*** | | | | | | |
| L/R medial frontal gyrus | 820 | 6 8 9 32 | 12.21 | 3 | 30 | 48 |
| L/R superior/middle frontal gyrus |  |  |  |  |  |  |
| L/R supplementary motor area |  |  |  |  |  |  |
| L/R middle cingulate cortex |  |  |  |  |  |  |
| R superior/middle frontal gyrus | 735 | 9 10 46 | 9.70 | 30 | 6 | 54 |
| R inferior frontal gyrus |  |  |  |  |  |  |
| R superior parietal lobule | 515 | 7 39 40 | 14.71 | 48 | -45 | 51 |
| R supramarginal/inferior parietal lobule |  |  |  |  |  |  |
| L superior parietal lobule | 386 | 7 40 | 10.24 | -39 | -51 | 45 |
| L supramarginal/inferior parietal lobule |  |  |  |  |  |  |
| R pre-cuneus | 191 | 7 | 8.69 | 9 | -75 | 48 |
| L middle frontal gyrus | 158 | 9 | 7.13 | -39 | 30 | 33 |
| L middle frontal gyrus | 155 | 9 10 | 9.46 | -33 | 48 | 9 |
| R inferior frontal gyrus | 148 | 13 47 | 8.83 | 33 | 21 | -6 |
| R insula |  |  |  |  |  |  |
| L inferior frontal gyrus | 104 | 13 47 | 7.30 | -33 | 18 | 6 |
| L insula |  |  |  |  |  |  |
| ***Free non-switching vs. forced non-switching*** | | | | | | |
| L superior/middle frontal gyrus | 497 | 6 8 9 46 | 9.08 | -42 | 27 | 33 |
| R supramarginal/inferior parietal lobule | 427 | 7 39 40 | 9.44 | 45 | -45 | 48 |
| R superior parietal lobule |  |  |  |  |  |  |
| L superior/inferior parietal lobule | 285 | 7 40 | 7.24 | -36 | -63 | 51 |
| L/R supplementary motor area | 278 | 6 8 32 | 9.02 | 0 | 27 | 48 |
| L/R medial frontal gyrus |  |  |  |  |  |  |
| L/R cingulate cortex |  |  |  |  |  |  |
| R superior/middle frontal gyrus | 237 | 6 8 | 7.08 | 27 | 6 | 57 |
| R middle frontal gyrus | 228 | 8 9 46 | 6.77 | 45 | 27 | 42 |
| L middle/inferior frontal gyrus | 207 | 10 11 46 | 10.43 | -39 | 51 | 6 |
| R inferior frontal gyrus | 98 | 13 47 | 7.67 | 33 | 21 | 3 |
| R insula |  |  |  |  |  |  |
| L inferior frontal gyrus | 85 | 13 47 | 7.91 | -30 | 18 | -3 |
| L insula |  |  |  |  |  |  |
| R middle frontal gyrus | 72 | 10 11 | 6.40 | 33 | 57 | 6 |
| L pre-cuneus | 53 | 7 | 7.26 | -9 | -69 | 48 |
| ***Forced non-switching vs. free non-switching*** | | | | | | |
| L/R anterior cingulate cortex | 1133 | 9 10 11 24 32 | 13.34 | -9 | 42 | 0 |
| L/R medial frontal gyrus |  |  |  |  |  |  |
| L/R superior frontal gyrus |  |  |  |  |  |  |
| L/R orbital frontal gyrus |  |  |  |  |  |  |
| L fusiform gyrus | 357 | 36 37 | 7.80 | -36 | -57 | -12 |
| L parahippocampa gyrus |  |  |  |  |  |  |
| L posterior cingulate cortex | 237 | 23 31 | 5.91 | -9 | -54 | 12 |
| L/R pre-cuneus |  |  |  |  |  |  |
| L middle occipital gyrus | 122 | 19 | 5.45 | -45 | -84 | 18 |
| L middle/inferior temporal gyrus | 101 | 21 | 6.79 | -54 | 0 | -21 |
| R middle temporal gyrus | 74 | 19 | 5.62 | 42 | -78 | 21 |
| R supramarginal gyrus | 70 | 40 | 7.16 | 69 | -30 | 33 |
| R fusiform gyrus | 69 | 20 37 | 6.52 | 39 | -39 | -18 |
| L inferior frontal gyrus | 58 | 47 | 8.50 | -33 | 30 | -15 |
| L superior temporal gyrus | 54 | 39 | 5.65 | -51 | -57 | 18 |

**p*<0.001, k>50 voxels, FDR-corrected

**Table S3. Activated Brain Regions during Forward Language Switching Versus Non-Switching and Backward Language Switching Versus Non-Switching***

| **Region** | **Cluster size** | **BA**  **areas** | **T**  **value** | **MNI coordinates** | | |
| --- | --- | --- | --- | --- | --- | --- |
|  |  |  |  | **x** | **y** | **z** |
| ***Free backward switching (into 1) vs. free L1 non-switching*** | | | | | | |
| L/R superior frontal gyrus | 421 | 6 9 10 32 | 8.33 | -6 | 45 | 30 |
| L/R medial frontal gyrus |  |  |  |  |  |  |
| L/R anterior cingulate cortex |  |  |  |  |  |  |
| ***Free L1 non-switching vs. free backward switching (into 1)*** | | | | | | |
| L pre/post-central gyrus | 330 | 3 4 40 | 8.48 | -36 | -21 | 51 |
| L inferior parietal lobule |  |  |  |  |  |  |
| R culmen | 72 | n/a | 6.79 | 24 | -57 | -27 |
| ***Forced backward switching (into 1) vs. forced L1 non-switching*** | | | | | | |
| L pre-central/post-central frontal gyrus | 755 | 3 4 6 8 32 40 | 8.39 | -48 | -18 | 48 |
| L superior frontal gyrus |  |  |  |  |  |  |
| L/R supplementary motor area |  |  |  |  |  |  |
| L/R middle cingulate cortex |  |  |  |  |  |  |
| L/R inferior parietal lobule |  |  |  |  |  |  |
| L/R cerebellum crus1 | 671 | n/a | 7.77 | 24 | -54 | -30 |
| L fusiform gyrus |  |  |  |  |  |  |
| L pre-cuneus | 213 | 7 18 19 | 7.84 | -24 | -72 | 30 |
| L superior occipital cortex |  |  |  |  |  |  |
| L superior parietal lobule |  |  |  |  |  |  |
| L thalamus | 200 | n/a | 8.79 | -12 | -15 | -12 |
| L caudate |  |  |  |  |  |  |
| L supramarginal gyrus | 61 | 40 | 6.46 | -54 | -39 | 24 |
| R middle frontal gyrus | 57 | 6 | 6.75 | 42 | 0 | 57 |
| ***Forced L1 non-switching vs. forced backward switching (into 1)*** | | | | | | |
| R pre-central/post-central frontal gyrus | 136 | 3 4 | 7.46 | 51 | -21 | 60 |

**p*<0.001, k>50 voxels, FDR-corrected

**Table S4. Means (and Standard Deviations) of Subjects' Self-Ratings and Proﬁciency T**ests

|  | **L1 (Chinese)** | **L2 (English)** |
| --- | --- | --- |
| ***Self-rating (LEPQ)*** |  |  |
| AoA |  | 11.9 (1.2) |
| Listening | 9.3 (0.4) | 8.4 (0.6) |
| Reading | 9.5 (0.3) | 8.7 (0.5) |
| Speaking | 9.2 (0.4) | 8.2 (0.5) |
| ***Proficiency test (TEM4)*** |  |  |
| Listening |  | 77% (5.7%) |
| Reading |  | 79% (6.2%) |
| Writing |  | 71% (5.2%) |

**Figures and Legends (Supplementary, Total 2)**

**
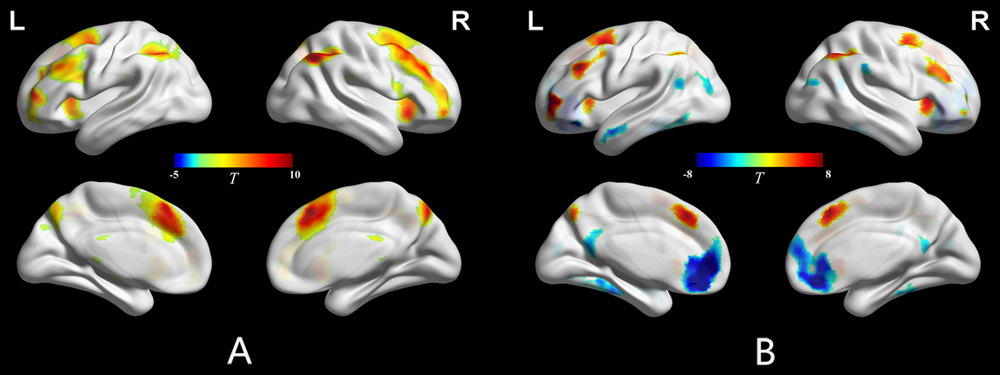
**

**Figure S1. Free Language Switching Versus Forced Language Switching and Free Language Non-Switching Versus Forced Language Non-Switching**

A. Results from the comparison of free language switching and forced language switching. Activations depicted in “hot” colors represent regions that were signiﬁcantly more active during free language switching as compared to forced language switching. Activations depicted in “cold” colors represent regions that were signiﬁcantly more active during forced language switching as compared to free language switching. B. Results from the comparison of free language non-switching and forced language non-switching. Activations depicted in “hot” colors represent regions that were signiﬁcantly more active during free language non-switching as compared to forced language non-switching. Activations depicted in “cold” colors represent regions that were signiﬁcantly more active during forced language non-switching as compared to free language non-switching. *p*<0.001, k>50 voxels, FDR-corrected.


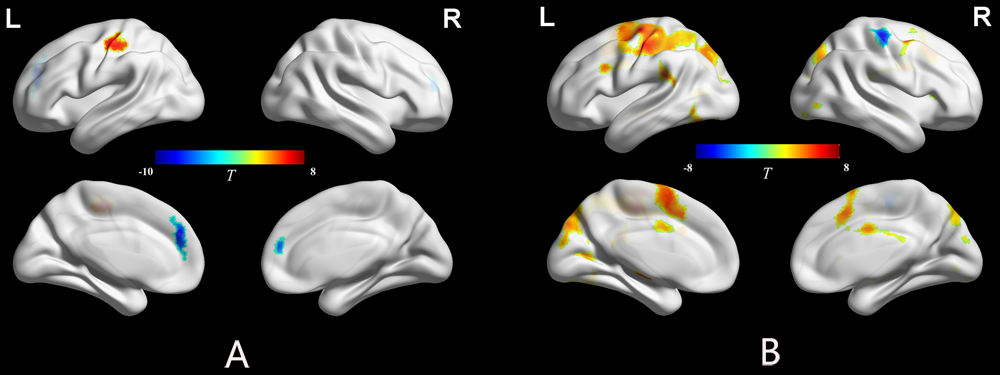


**Figure S2. Free Backward Switching (into L1) Versus Free L1 Non-Switching and Forced Backward Switching (into L1) Versus Forced L1 Non-Switching**

A. Results from the comparison of free backward switching (into L1) and free L1 non-switching. Activations depicted in “hot” colors represent regions that were signiﬁcantly more active during free backward switching as compared to free L1 non-switching. Activations depicted in “cold” colors represent regions that were signiﬁcantly more active during free L1 non-switching as compared to free backward switching. B. Results from the comparison of forced backward switching (into L1) and forced L1 non-switching. Activations depicted in “hot” colors represent regions that were signiﬁcantly more active for forced backward switching as compared to forced L1 non-switching. Activations depicted in “cold” colors represent regions that were signiﬁcantly more active during forced L1 non-switching as compared to forced backward switching. *p*<0.001, k>50 voxels, FDR-corrected.
